# Arginine metabolism in myeloid cells in health and disease

**DOI:** 10.1007/s00281-025-01038-9

**Published:** 2025-01-25

**Authors:** Eleftheria Karadima, Triantafyllos Chavakis, Vasileia Ismini Alexaki

**Affiliations:** Institute for Clinical Chemistry and Laboratory Medicine, Faculty of Medicine and University Hospital Carl Gustav Carus, Technische Universität Dresden, Fetscherstrasse 74, 01307 Dresden, Germany

**Keywords:** Arginine metabolism, Myeloid cells, Infection, Inflammation, Cancer, Sepsis-induced immune paralysis, Surgery

## Abstract

Metabolic flexibility is key for the function of myeloid cells. Arginine metabolism is integral to the regulation of myeloid cell responses. Nitric oxide (NO) production from arginine is vital for the antimicrobial and pro-inflammatory responses. Conversely, the arginase 1 (ARG1)-dependent switch between the branch of NO production and polyamine synthesis downregulates inflammation and promotes recovery of tissue homeostasis. Creatine metabolism is key for energy supply and proline metabolism is required for collagen synthesis. Myeloid ARG1 also regulates extracellular arginine availability and T cell responses in parasitic diseases and cancer. Cancer, surgery, sepsis and persistent inflammation in chronic inflammatory diseases, such as neuroinflammatory diseases or arthritis, are associated with dysregulation of arginine metabolism in myeloid cells. Here, we review current knowledge on arginine metabolism in different myeloid cell types, such as macrophages, neutrophils, microglia, osteoclasts, tumor-associated macrophages (TAMs), tumor-associated neutrophils (TANs) and myeloid-derived suppressor cells (MDSCs). A deeper understanding of the function of arginine metabolism in myeloid cells will improve our knowledge on the pathology of several diseases and may set the platform for novel therapeutic applications.

## Introduction

Myeloid cells have multiple functions including elimination of microbes, antigen presentation, cytokine secretion, T cell activation, resolution of inflammation and tissue repair [1]. The functional versatility of myeloid cells is enabled by their substantial metabolic plasticity. Particularly arginine metabolism is highly flexible and plays a key role in the modulation of immune function [2]. Almost 100 years after the discovery of the urea cycle in 1932 [3], the importance of arginine metabolism in the regulation of innate and adaptive immune responses is still an area of interest. Here, we review current knowledge on arginine metabolism and its role in myeloid cell function in the context of infection, sepsis, chronic inflammatory disease, cancer and surgery.

## Myeloid cells

The myeloid cell compartment constitutes a major pillar of innate immunity. It comprises granulocytes, monocytes, dendritic cells, tissue macrophages and mast cells [4]. Granulocytes, monocytes, and dendritic cells derive from bone marrow hematopoietic stem and progenitor cells, while most tissue-resident macrophages, such as microglia, Kupffer cells or Langerhans cells, originate from embryonic precursors of the yolk sac or the fetal liver [1]. Tissue-resident myeloid cells have long life spans and perform homeostatic functions [1]. In contrast, bone marrow-derived myeloid cells are mobilized upon trauma or infection [1, 5]. Neutrophils are the first line of defense against pathogens. They are rapidly recruited to infected sites, and eliminate microbes through phagocytosis, antimicrobial substances, reactive oxygen species (ROS) and formation of neutrophil extracellular traps (NETs) [6, 7]. Tissue-resident macrophages are replenished by bone marrow monocyte-derived macrophages during life [4]. Upon infection or trauma, monocytes infiltrate affected tissues and transform to inflammatory macrophages, which contribute to elimination of pathogens [4]. Moreover, macrophages are central to mediating resolution of inflammation by clearing dead cells, which triggers restoration of tissue integrity [8, 9]. Dendritic cells play a unique role in bridging the innate and adaptive immune responses [10]. They are localized at sites of possible pathogen entry in order to rapidly detect and phagocytose microbes, and to stimulate T cells through antigen presentation, thereby priming the adaptive immune response [10]. Eosinophils respond to helminth infection and allergen exposure by releasing positively charged proteins stored in cytoplasmic granules [5]. Basophils are the rarest and least studied granulocytes. They are implicated in allergic reactions, immunity against parasitic infections, autoimmunity and tissue restoration [11]. Metabolic circuits regulating immune responses, including arginine metabolism, are mostly studied in monocytes / macrophages due to their relative abundance and easy isolation and culture, while in some myeloid cell types, such as eosinophils or mast cells, knowledge on cell metabolic regulation is scarce.

## Arginine metabolism in myeloid cells

**Fig. 1:**
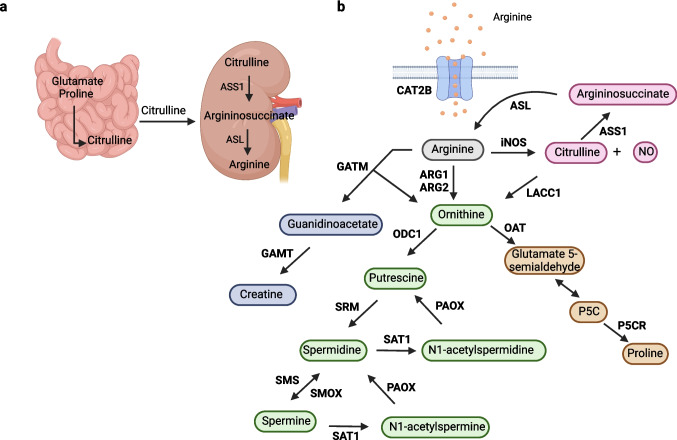
Arginine metabolism in myeloid cells. **a**. Arginine biosynthesis. Arginine is synthesized in humans in the intestinal-renal axis. Citrulline is produced by glutamate or proline in enterocytes and taken up by the kidney, where it is transformed to argininosuccinate through argininosuccinate synthase 1 (ASS1), which is subsequently metabolized to arginine by argininosuccinate lyase (ASL) [2]. **b**. Arginine metabolism in myeloid cells. Arginine is transported into the cells via CAT-2B and metabolized to ornithine through the function of arginases 1 or 2 (ARG1, ARG2). Ornithine is then converted to putrescine through the function of ornithine decarboxylase 1 (ODC1) [2, 15, 16]. Putrescine is further converted through spermidine synthase (SRM) to spermidine, which is metabolized by spermine synthase (SMS) to spermine [2]. Spermine can be converted back to spermidine via oxidation. Also, polyamines undergo acetylation through spermidine/spermine-N1-acetyltransferase (SAT1) to N1-acetyl-spermidine and N1-acetyl-spermine, which are then oxidized via polyamine oxidase (PAOX) to their respective precursors [19]. Upon inflammation, arginine is diverted towards the production of citrulline and nitric oxide (NO) through inducible nitric oxide synthase (iNOS) [2, 17]. Citrulline can be also converted to ornithine by laccase domain-containing 1 (LACC1), the expression of which increases under inflammatory conditions [20]. Moreover, ornithine can be metabolized to proline through L-glutamate 5-semialdehyde and 1-pyrroline 5-carboxylate (P5C) [2]. Furthermore, arginine can be metabolized to creatine through the production of guanidinoacetate and the action of glycine amidinotransferase (GATM) and guanidinoacetate N-methyltransferase (GAMT) [2]. The figure was created with BioRender.com

Arginine metabolism presents substantial differences between different cell types and species. In humans, arginine is synthesized in the intestinal-renal axis. Citrulline is produced in enterocytes from glutamate or proline, and taken up by the kidney, where it is converted to arginine via argininosuccinate through the function of argininosuccinate synthase (ASS1) and argininosuccinate lyase (ASL) (Fig. 1a). However, under extreme conditions, such as critical illness or maximal growth during infancy, arginine becomes an essential amino acid and must be obtained through the diet [12]. Interestingly, macrophages acquire the ability to produce arginine from citrulline upon inflammatory activation [13, 14].

Circulating arginine from the diet and/or endogenous synthesis is transported into the cells mostly by the solute carrier 7 family (SLC7) members cationic amino acid transporters CAT-1 and CAT-2 [15, 16]. Macrophages mainly express CAT-2B (encoded by *Slc7a2*), which is induced upon classical or alternative activation, and exhibits high affinity for arginine [15, 16]. The importance of arginine metabolism in macrophage immune responses is demonstrated in *Slc7a2*^*−/−*^ mice, which have reduced antimicrobial resistance [16]. Intracellular arginine is either used for protein synthesis or metabolized [17, 18]. It can be converted by arginases 1 or 2 (ARG1, ARG2) to ornithine, which is then metabolized via ornithine decarboxylase 1 (ODC1) to putrescine. The latter is a substrate of spermidine synthase (SRM) producing spermidine, which is further converted to spermine by spermine synthase (SMS). Spermine can be converted back to spermidine via oxidation. Furthermore, polyamines are acetylated by spermidine/spermine-N1-acetyltransferase (SAT1) to N1-acetyl-spermidine and N1-acetyl-spermine, which can be oxidized by polyamine oxidase (PAOX) to regenerate their precursor molecules [2, 18, 19]. Ornithine is further metabolized to proline through L-glutamate 5-semialdehyde and 1-pyrroline 5-carboxylate (P5C) [2, 18]. Arginine is also metabolized via L-Arginine:glycine amidinotransferase (GATM) using glycine to ornithine and guanidinoacetate, the latter being further metabolized to creatine through the action of guanidinoacetate N-methyltransferase (GAMT) [2] (Fig. 1b). Polyamine metabolism, proline synthesis and creatine synthesis contribute to tissue homeostasis or promote tissue recovery after injury or infection.

**Fig. 2:**
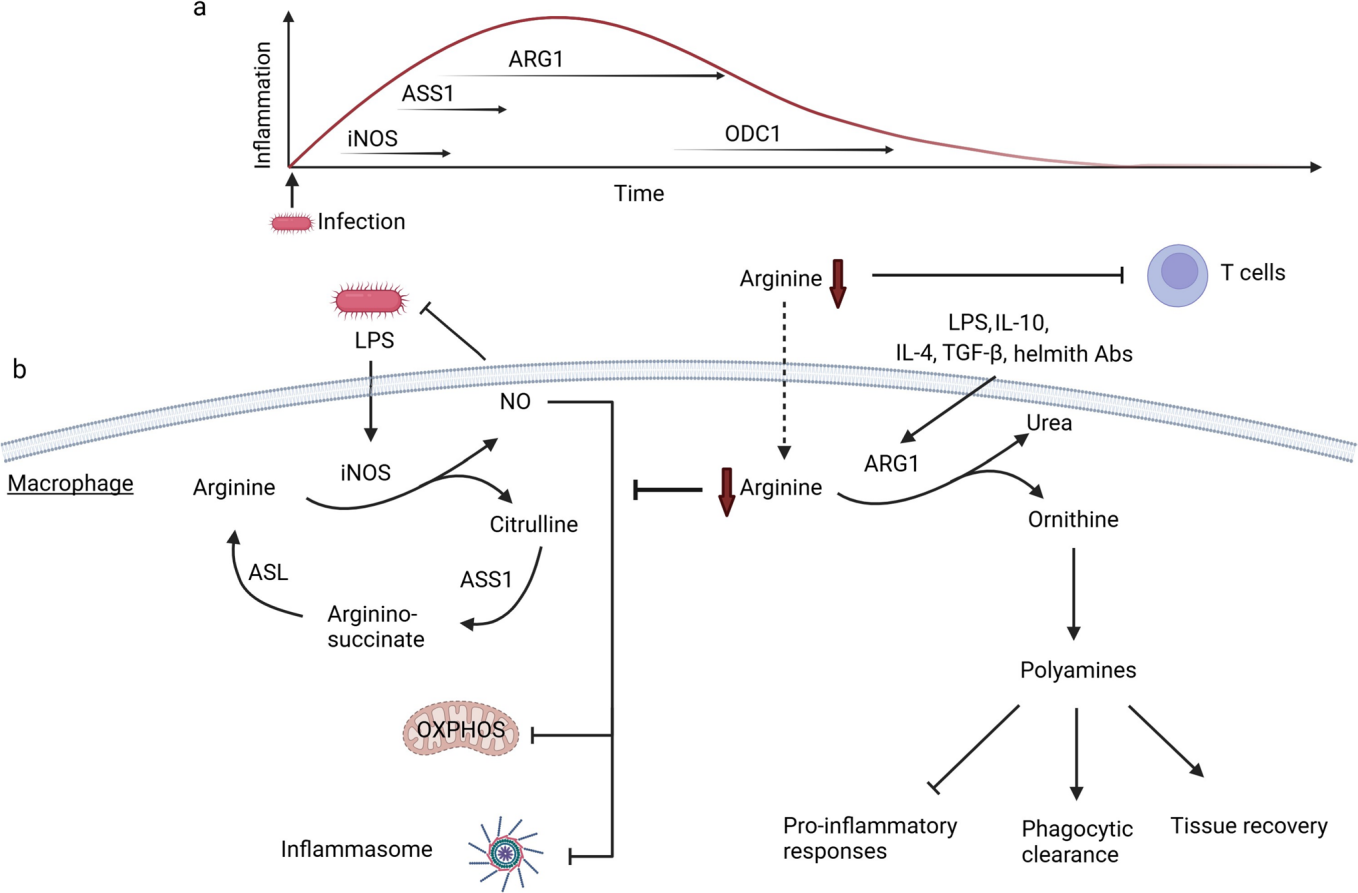
The function of arginine metabolism in macrophage inflammatory responses. **a**. Changes in arginine metabolism in the course of inflammation. In the early inflammatory response, iNOS is induced [2]. ASS1 is upregulated to sustain iNOS function [13]. Later, ARG1 is upregulated to limit iNOS function [17, 56] and shift arginine metabolism toward production of polyamines, which may contribute to termination of inflammation [2, 100, 101]. **b**. Pathways of arginine metabolism regulating macrophage immune responses. Bacteria, LPS and IFNγ induce iNOS, resulting in production of NO, which is toxic for bacteria [13, 37]. NO also disrupts oxidative phosphorylation [44] and inhibits inflammasome activation in macrophages [47, 48]. Induction of the ASS1- and ASL-mediated argininosuccinate shunt sustains the function of iNOS through production of arginine [13]. ARG1 competes with iNOS for arginine, thereby inhibiting its function [17, 38, 57, 62]. ARG1 expression is induced by different pro- and anti-inflammatory stimuli, such as by LPS, IL-10, IL-4, TGF-β and helminth antibodies [56, 57, 65, 67, 69, 72]. ARG1 is mostly dependent on extracellular arginine, thereby regulating arginine availability to other cells, such as T cells [17, 18, 76]. Ornithine, the product of ARG1, is the precursor of polyamines, which inhibit inflammation and contribute to tissue remodeling [2, 92, 100, 101, 134]. The figure was created with BioRender.com

Trauma and pathogens trigger shunting of arginine to production of citrulline and nitric oxide (NO) through the function of the inducible nitric oxide synthase (iNOS) [2, 13, 17] (Fig. 2a). In prolonged inflammation, due to arginine shortage, macrophages acquire the ability of arginine biosynthesis by converting citrulline via ASS1 to argininosuccinate, which is further metabolized via ASL to arginine [13]. Finally, citrulline can be also converted to ornithine by laccase domain-containing 1 (LACC1), the expression of which increases upon inflammation in macrophages [20] (Fig. 1b).

Numerous studies have addressed the roles of iNOS and ARG1 in macrophage polarization [2, 17]. The latter refers to the differential response of macrophages to in vitro stimulation with the Toll-Like Receptor 4 (TLR4) agonist lipopolysaccharide (LPS) and interferon-γ (IFNγ) or the type 2 immunity-related cytokines Interleukin 4 (IL-4) and IL-13 [21]. LPS or IFNγ induce classical activation hallmarked by production of inflammatory cytokines, ROS, a strong induction of iNOS expression and shift of glucose metabolism to glycolysis (Warburg-like effect) [21]. IL-4 induces alternative activation, characterized by elevated expression of scavenger receptors, matrix remodeling factors, and a strong induction of ARG1 expression [21]. Classical activation is considered to represent the pro-inflammatory response of macrophages to infection or trauma, while alternative activation is generally associated with type 2 immune responses (response to parasite infections, allergies, asthma), and reparative, wound healing responses [21]. However, in vitro macrophage polarization does not reflect the in vivo complexity, which is characterized by location-, origin- and function-related heterogeneity of macrophages driven by exposure to multiple local and systemic signals [21, 22]. Despite the limited relevance of in vitro macrophage polarization to the in vivo reality and pathology of inflammatory disease, it has been a useful tool in order to dissect the role of cell metabolism, and arginine metabolism in particular, in macrophages.

## Myeloid arginine metabolism in healthy conditions versus disease

Resident myeloid cells, including microglia (macrophage-like cells of the brain), Kupffer cells (macrophages of the liver), lung macrophages and Langerhans cells (skin macrophages), preserve tissue homeostasis by removing dead cells and debris and maintaining tissue architecture through extracellular matrix deposition [1]. In healthy conditions, polyamine, proline and creatine metabolism are sustained at relatively low levels, while citrulline is not produced. However, upon infection, trauma or disease, all pathways of arginine metabolism, i.e. citrulline, polyamine, creatine and proline production, are upregulated in an orchestrated time-dependent manner to support the antimicrobial function of myeloid cells, supply energy, facilitate phagocytosis, and promote tissue recovery.

## The role of iNOS in macrophage inflammatory responses

Bacterial infection or stimulation with LPS, IFNγ and/or cytokines triggers iNOS expression in human and mouse macrophages [13, 23–26]. In contrast to the relatively short promoter region of the murine *Nos2* gene [27, 28], the human *NOS2* gene is regulated by a classical enhancer region, located between 5 and 6 kb upstream of the transcription site, containing functional binding sites for nuclear factor kappa B (NFκB) and Signal transducer and activator of transcription 1 (STAT1) [29, 30]. Regulation of iNOS mostly occurs at the transcriptional level, but post-transcriptional modifications may also alter its expression [31].

In murine macrophages, iNOS generates large amounts (at the micromolar range) of NO, while production of NO in human macrophages is under debate [13, 23–25, 32, 33]. Unlike inflammatory macrophages, neutrophils produce little NO [34]. NO is toxic and can directly kill bacteria but also suppresses neutrophil recruitment [13, 35]. NOS deficient mice are highly susceptible to *Mycobacterium tuberculosis* (Mtb) infection and develop severe lung and spleen pathology [24, 36]. However, NO is not always sufficient to restrain bacterial infection [24, 25]. For instance, NO produced by human macrophages does not sufficiently limit mycobacterial replication or survival [25]. Aside from its function in elimination of bacteria, NO exerts numerous functions within macrophages and their surrounding microenvironment [37].

As inflammation progresses, consumption of arginine used for NO synthesis in macrophages as well as arginine consumption by T cells, leads to arginine shortage. Arginine availability is required for iNOS expression and function [38]. Under conditions of limited arginine availability, macrophages regenerate arginine to sustain NOS production throughout the course of the inflammatory response [13]. Citrulline, which is exported from macrophages at early stages of inflammation, is imported at later stages to sustain NO production via activating ASS1/ASL-dependent arginine synthesis [13]. The importance of arginine regeneration in macrophages is evidenced by the fact that mice bearing hematopoietic cell-specific ASS1 deficiency are more sensitive to Mtb infection compared to wild-type control mice [13]. Accordingly, myeloid deficiency of ASS1 or ASL increases the *Mycobacterium bovis bacillus*, *Calmette-Guerin* and Mtb infection burden [39]. The aspartate-argininosuccinate shunt also affects the tricarboxylic acid (TCA) cycle flux through the production of fumarate, which accumulates in proinflammatory macrophages [40].

In macrophages, NO disrupts the function of the electron transport chain (ETC) through inhibition of the catalytic NADH binding module of complex I and complex IV (cytochrome c oxidase) [23, 41–44]. Moreover, NO regulates TCA cycle function by decreasing pyruvate dehydrogenase (PDH) and aconitase (ACO2) activity, reducing succinate and itaconate levels and increasing citrate [23, 44]. NO-mediated reduction of PDH and ACO2 enzymatic activities leads to a lower NADH/NAD^+^ ratio, which in turn promotes the conversion of complex I to its inactive form due to substrate deprivation [44]. Furthermore, NO may cause cysteine nitrosylation of numerous proteins, including enzymes involved in the TCA cycle, oxidative phosphorylation, glycolysis, gluconeogenesis and fatty acid metabolism; NO may thus regulate several metabolic reactions [45]. Consequently, NO downregulates mitochondrial respiration in inflammatory macrophages and mediates reprograming of macrophage cell metabolism from oxidative phosphorylation to glycolysis [14, 44, 46]. Accordingly, inhibition of arginine regeneration through the aspartate-argininosuccinate shunt inhibits NO production and inflammatory activation of macrophages and enhances oxidative phosphorylation [14] (Fig. 2b). Moreover, iNOS inhibition not only increases mitochondrial respiration in LPS + IFNγ treated macrophages, but also enhances M2-like polarization after a subsequent IL-4 treatment [43]. An NO-driven metabolic switch towards glycolysis also occurs in inflammatory dendritic cells [46]. Nevertheless, NO deficiency does not hinder upregulation of glycolysis in inflammatory macrophages suggesting implication of NO-independent mechanisms in the glycolytic shift of the cells [23].

Genetic deletion or pharmacological inhibition of iNOS suppress disease in a mouse psoriasis model due to reduced IL-1α production by skin macrophages [33]. However, iNOS deficient macrophages produce higher amounts of IL-1β and IL-6 than wild-type macrophages upon LPS stimulation, while production of TNF and IL-10 is not affected [23, 35, 44]. NO-mediated S-nitrosylation of NLRP3 prevents inflammasome activation thereby reducing IL-1β production [47]. NO may also synergize with itaconate to block NLRP3 inflammasome activation [48]. Through repression of IL-1 signaling, NO inhibits neutrophil recruitment after Mtb infection [35, 47]. Moreover, NO inhibits NF-κB activity thereby preventing overt inflammation upon Mtb infection [49] (Fig. 2b). By contrast, NO was proposed to stabilize HIF-1α [49], which in turn promotes IL-1β production [50]. Collectively, these data demonstrate the complexity of NO-mediated regulation of inflammatory pathways in macrophages.

iNOS requires tetrahydrobiopterin (BH4) as a cofactor to produce NO [23]. Interestingly, iNOS and BH4 do not always share common effects. For instance, BH4 deficient macrophages have enhanced control of mycobacterial infection, while iNOS deficient macrophages present reduced responses against mycobacteria [24]. The differential impact of BH4 and iNOS deficiency on macrophage function following Mtb infection is reflected by distinct changes in the transcriptomic landscape of macrophages [24]. However, NOS enzymes can also function independently of BH4 and NO production through their reductase activity producing superoxide [51]. This is of particular relevance for human macrophages, which have low amounts of BH4 [24].

## The role of arginases in myeloid cells

Inflammation comes with collateral damage of the host tissue. NO is toxic not only for bacteria, but also for host cells including macrophages themselves. As a means to restrain NO production, macrophages upregulate arginases [17, 52] (Fig. 2a, b). For instance, macrophages in granulomas in human tuberculosis express both, iNOS and ARG1 [53]. *Arg1* expression is controlled by an enhancer region 3 kb upstream of the transcription site. PU.1, STAT6, and C/EBP bind at the enhancer region and promote *Arg1* expression [54, 55]. Gram-negative bacteria or LPS trigger ARG1 expression in macrophages [56, 57]. Mycobacterium-infected macrophages produce IL-6, IL-10 and granulocyte colony-stimulating factor (G-CSF) that sustain ARG1 expression in an autocrine and paracrine manner via STAT3 signaling [52, 58]. In alternatively activated macrophages, STAT6 signaling promotes *Arg1* expression [58]. Moreover, IL-10 can synergistically with LPS induce ARG1 expression in macrophages [59]. LPS also triggers negative feedback mechanisms, such as the expression of the transcription factor Fra-1 (Fos-related antigen 1), to suppress macrophage ARG1 expression [60].

Many studies suggest that ARG1 may compete with iNOS for arginine thereby limiting iNOS function [17, 38, 57, 61–63]. The temporal pattern of iNOS and ARG1 expression reflects their function during the course of inflammation: iNOS protein levels peak at 12 h, whereas ARG1 protein expression increases 24 h after induction of M1-like polarization [56] (Fig. 2a). Mechanistically, it was shown that glycolysis-derived lactate mediates lactylation of histone lysine residues in the *Arg1* gene promoter thereby increasing ARG1 expression in M1-like macrophages [56]. Consequently, ARG1-deficient inflammatory macrophages produce higher amounts of NO, and myeloid ARG1 deficiency decreases the lung bacterial load during Mtb infection [52]. ARG1 preferentially uses arginine imported from the extracellular milieu rather than arginine synthesized in macrophages thereby regulating arginine availability to neighboring cells [13, 64] (Fig. 2b).

During parasite infection, activated T cells and eosinophils induce ARG1 expression in macrophages through production of IL-4 and IL-13 and activation of STAT6 [55, 65, 66] (Fig. 2b). However, other cytokines involved in resolution of inflammation and wound healing do also increase ARG1 expression: for instance, TGF-β increases ARG1 in macrophages and dendritic cells [67, 68] and IL-10 upregulates the expression of the IL-4 receptor α (IL-4Rα) thereby acting synergistically with IL-4 to promote ARG1 expression [69, 70]. In contrast, TNF inhibits IL-4-induced ARG1 expression and TNF deficiency in *Leishmania major* infected mice leads to ARG1 hyperexpression in myeloid cells, impaired NO production and increased lethality [62]. Moreover, neutrophils may promote ARG1 expression in macrophages upon parasite infection, leading to reduced helminth survival due to arginine depletion [71]. Helminth-specific antibodies can also induce ARG1 in macrophages that subsequently promotes the trapping of parasite larvae [72]. However, some parasites, such as *Leishmania*, induce ARG1-driven arginine metabolism in macrophages and dendritic cells in order to hijack myeloid polyamine production and thereby to support their own intracellular growth [73, 74]. Despite these evolutional adaptations serving parasite survival, ARG1-expressing macrophages are in general designed to fight parasite infection by walling off toxic worm eggs through collagen deposition enabling their disposal [17]. The pro-fibrotic effect of ARG1 may be due to promotion of proline synthesis, which is required for collagen production [75]. Yet, mice with myeloid ARG1 deficiency display increased collagen deposition upon *Schistosoma mansoni* (*S. mansoni*) infection [65], indicating the complex regulation of fibrosis in the setting of type 2 immunity.

L-arginine is unconditionally needed for T cell activation [76]. The expression of arginase isoforms in T cells appears to be context-dependent [18, 77]. For instance, while arginine is mainly metabolized by ARG2 in tumor-associated T cells [18], it is metabolized by ARG1 in lung CD4^+^ T cells upon influenza infection [77]. High arginase activity in myeloid cells results in extracellular arginine depletion causing T cell hyporesponsiveness [76, 78, 79] (Fig. 2b). Consequently, *S. mansoni*-infected mice with myeloid-specific ARG1 deficiency develop lethal non-resolving T cell-driven inflammation, suggesting that myeloid ARG1 controls Th2-dependent responses by depriving T cells from arginine [17, 65, 78, 80]. However, the effects of myeloid ARG1 are tissue-, disease- and context-dependent. For instance, macrophage ARG1 deficiency does not alter T cell-driven asthma pathogenesis in mice [81]. Moreover, inhibition of both ARG1 and iNOS exacerbates lung granuloma pathology and the bacterial burden in Mtb infected mice, possibly due to uncontrolled Th1 cell responses [82], while *S. mansoni*-induced ARG1 exacerbates lung inflammation upon co-infection with Mtb [83].

In contrast to cytosolic ARG1, the function of mitochondrial ARG2 in myeloid cells has received less attention [59]. Similarly to ARG1 expression, ARG2 expression is upregulated in human and mouse macrophages upon LPS treatment [59]. ARG2 increases mitochondrial respiration by promoting succinate dehydrogenase (complex II) activity, thereby downregulating succinate levels and consequently Hypoxia Inducible Factor 1 Subunit Alpha (HIF-1a) and IL-1β expression, and promoting an anti-inflammatory phenotype in macrophages [59, 84, 85]. However, proinflammatory effects of ARG2 for instance through increase of mitochondrial ROS, were also reported [86].

In conclusion, during tissue repair or parasite infection, whereby synthesis of extracellular matrix and overall anabolism is of paramount importance, it seems counterintuitive that macrophages upregulate arginine catabolism, while also increasing its uptake. This seeming paradox can be explained by the necessity of 1. arginine depletion by ARG1 to dampen iNOS activity and thereby avoid toxic NO overproduction, 2. deprivation of T cells from arginine thereby controlling T cell responses, and 3. production of arginine metabolites, such as polyamines and proline, which may contribute to regulation of inflammation and tissue remodeling (Fig. 2a, b).

## The role of polyamines in regulation of inflammatory responses

Production of polyamines (putrescine, spermidine and spermine) starts with ornithine decarboxylation, i.e. the rate-limiting step of polyamine synthesis [19]. In inflammatory macrophages, ornithine derives from both arginine via ARG1 and ARG2 and citrulline via LACC1 [2, 20]. LACC1 deficient mice have exacerbated inflammation upon *S. Typhimurium* infection, which is reversed by ornithine treatment [20]. ODC1 myeloid deficiency increases gastric and colonic inflammation upon *Helicobacter pylori* and *Citrobacter rodentium* infection, respectively, while putrescine reverses these effects [87, 88]. Moreover, bacterial putrescine increases the abundance of anti-inflammatory macrophages and promotes mucosal homeostasis in the intestine of mice with dextran sulfate sodium (DSS)-induced colitis [89]. Similar to ARG1, the anti-inflammatory effect of ODC1 is associated with inhibition of iNOS [88, 90]. Mechanistically, a driver of polyamine synthesis in macrophages is mammalian target of rapamycin complex 1 (mTORC1) [91].

Polyamines dampen macrophage inflammatory responses and promote macrophage alternative activation through several mechanisms [20, 92, 93]. Spermidine can be transformed to hypusine by the deoxyhypusine synthase (DHPS). Hypusine is an uncommon amino acid present only in a single protein, the translation factor eukaryotic initiation factor 5A (eIF5A), and is crucial for eIF5A function. Hypusination of eIF5A increases the expression of mitochondrial proteins participating in the TCA cycle and oxidative phosphorylation, and promotes alternative activation of macrophages [94]. Spermidine also binds to mitochondrial trifunctional protein (MTP), promoting fatty acid oxidation, mitochondrial function and anti-tumoral activity of T cells [95]. Furthermore, polyamines promote autophagy, a homeostatic degradation and recycling mechanism of intracellular components, involved in cell survival and longevity [96, 97], which also impedes macrophage inflammatory responses and promotes alternative activation [98]. In addition, intracellular spermine inhibits K^+^ efflux-dependent NLRP3 inflammasome activation upon *Edwardsiella piscicida* infection [99].

Polyamine metabolism is also implicated in efferocytosis, i.e. phagocytosis of apoptotic cells, a process that contributes to resolution of inflammation. Polyamine levels increase in efferocytic macrophages, mainly via import [100, 101]. Putrescine promotes efferocytosis by increasing the expression of the guanosine triphosphate (GTP)-exchange factor (GEF) Dbl, which in turn activates Rac1 and actin polymerization enabling efferocytosis [101]. Consistently, ODC1 deficiency impairs continual efferocytosis and resolution of atherosclerosis, while putrescine restrains atherosclerosis [101]. Apoptotic cells release polyamines, which are uptaken by efferocytic macrophages, in parts via Rac1-dependent endocytic import [100, 102]. Received polyamines contribute to transcriptional reprogramming of macrophages leading to suppression of proinflammatory responses [100, 102]. Polyamines also promote wound healing and tissue remodeling [103–105]. In addition, ornithine released by arginase-expressing macrophages can be uptaken by fibroblasts and used for proline synthesis, which is required for collagen deposition [106].

## The role of proline metabolism in myeloid cell responses

Proline metabolism is upregulated in pro-fibrotic states [107] and collagen synthesis depends on proline availability [108]. Nevertheless, the role of proline metabolism in macrophages is poorly understood. A recent study revealed that Prolidase or Peptidase D (PEPD), which mediates collagen turnover by degrading proline-containing dipeptides and is expressed in adipose tissue macrophages, regulates adipose tissue fibrosis. PEPD expression and activity is reduced in the adipose tissue of obese humans and mice and its pharmacological inhibition or genetic deletion promotes adipose tissue fibrosis and insulin resistance. Interestingly, PEPD is secreted by adipose tissue macrophages, and extracellular PEPD increases inflammation and fibrosis in the adipose tissue by activating Epidermal Growth Factor Receptor (EGFR) signaling. Reduced prolidase activity in the adipose tissue correlates with increased circulating PEPD levels, which serve as a biomarker of adipose tissue inflammation and fibrosis [109]. Furthermore, proline metabolism is implicated in trained innate immunity in lung macrophages. The expression of the rate-limiting enzyme of proline biosynthesis, glutaminase (GLS), is upregulated in trained macrophages in an allergic asthma mouse model with respiratory virus infection and early life allergen sensitization. Inhibition of proline synthesis suppresses trained macrophages and the development of allergic asthma, while proline supplementation recovers trained macrophages [110]. Finally, proline catabolism in *Candida albicans* cells was shown to be essential for its hyphal growth in phagosomes of engulfing macrophages, a process that *C. albicans* uses to escape killing by macrophages [111].

## The role of creatine metabolism in macrophages

ATP-mediated energy supply is highly dependent on creatine metabolism [112]. Creatine is primarily synthesized in the liver and kidneys [113]. It is phosphorylated to creatine phosphate, which serves as a phosphate donor in the conversion of ADP to ATP, and thus supplies locally and acutely energy [114]. In macrophages, creatine is uptaken through the SLC6A8 transporter and phosphorylated by the cytosolic creatine kinase (CK-B) [115]. Exogenous creatine dampens IFNγ-triggered inflammatory responses, while it increases IL-4-induced responses, possibly through promoting ATP-dependent chromatin remodeling processes [115]. GATM expression is upregulated upon IL-4 treatment and is required for IL-4-induced responses [116]. In contrast, GATM expression is suppressed upon LPS treatment but does not affect proinflammatory macrophage responses [116]. In microglia, cyclocreatine, a membrane-transpassing creatine analogue, promotes microglial protective functions, suppresses autophagy and improves Aβ plaque-associated neurite destruction in a model of Alzheimer’s disease (AD) [117]. Mechanistically, CK-B is mobilized and interacts with F-actin at nascent phagosomes promoting actin polymerization and phagocytosis by increasing ATP supply in phagocytic macrophages [118]. This makes creatine metabolism crucial for the clearing function of macrophages.

## The role of arginine metabolism in sepsis-induced immunodeficiency

Sepsis is a dysregulated systemic inflammatory and immune response to a disseminated infection that leads to immune paralysis and organ dysfunction [119]. It is manifested by a cytokine storm, i.e. derailed production of pro- and anti-inflammatory cytokines, accompanied or followed by immune paralysis with prominent lymphopenia and impaired T cell numbers and function [119]. The mechanisms underlying sepsis-induced immunossuppression are not entirely understood. Given the role of arginine in T cell function, reduced arginine availability during sepsis may contribute to immune dysfunction. Despite the increased arginine release from catabolic protein breakdown, arginine plasma concentration is reduced in sepsis due to decreased de novo arginine synthesis and its increased consumption through myeloid ARG1 and iNOS [120, 121]. Consequently, limited arginine availability in sepsis may lead to impaired T cell proliferation and function and related immunosuppression [122]. Therapeutic citrulline administration increasing arginine plasma levels, restores T cell mitochondrial function and proliferation and reduces sepsis-induced immunosuppression [123]. Especially patients with persistent immunosuppressed inflammatory catabolic state (PICS) may benefit from arginine supplementation [124]. Hence, restoring arginine levels may be useful against sepsis-induced lymphopenia and immune paralysis.

## Myeloid arginine metabolism in chronic inflammatory diseases

### Microglia-mediated neuroinflammation

Microglia are resident immune cells of the central nervous system (CNS). They derive from embryonic yolk sac precursors and can self-renew. They perform various functions, including phagocytosis, synaptic pruning during neuronal development and inflammatory reactions to infection or injury [125]. In the healthy mouse brain, ARG1 is expressed in microglia of certain brain regions, such as the basal forebrain [126]. ARG1 expression is induced by IL-4Rα and STAT6 signaling in microglia and promotes their phagocytic capacity [126–128]. Microglia-specific ARG1 deficiency results in impaired neural maturation in the hippocampus, which consequently causes cognitive deficits in mice [126]. In mouse models of AD, a neurodegenerative disease characterized by deposition of amyloid beta peptide (Aβ), an early toxic event in AD pathogenesis, ARG1^+^ microglia mediate Aβ plaque clearance, while ARG1 overexpression in the CNS reduces inflammation and hippocampal atrophy and ameliorates disease [129, 130]. Moreover, microglia may release ARG1, which catabolizes extracellular arginine and reduces arginine levels in the brain [131]. However, inhibition of arginine catabolism with ARG1 blockers reduces microglial inflammation and mitigates AD-like pathology [131]. As in macrophages, arginine metabolism in microglia is reprogramed depending on the inflammatory state and phagocytic activity [126, 132]. For instance, upon demyelination, microglial cells highly express iNOS, while at later stages of remyelination they acquire an iNOS^−^/ARG1^+^ phenotype [132]. iNOS may promote citrullination of myelin, which impairs remyelination and increases neuroinflammation [133]. Hence, the switch from iNOS to ARG1 is essential for resolution of neuroinflammation and restoration of neural tissue integrity.

Upregulation of ARG1 expression leads to increased polyamine production [134]. Alterations in brain polyamine metabolism are associated with neurodegenerative disease pathology [135, 136]. Inflammation upregulates ODC1 expression in microglia and neurons [137]. In turn, spermidine counteracts microglia-mediated neuroinflammation and exerts neuroprotective effects [138, 139]. Spermidine administration increases expression of genes related to phagocytosis in microglia and reduces Aβ pathology in an AD mouse model [138]. Moreover, administration of spermidine to mice with experimental autoimmune encephalomyelitis (EAE), a model of multiple sclerosis, the most prevalent demyelinating disease in humans, diminishes the potential of macrophages for T cell activation and ameliorates disease severity [139].

### Arthritis

Osteoclasts are multinucleated giant cells derived from myeloid precursors. They regulate bone homeostasis through bone resorption, as opposed to bone-forming osteoblasts. Osteoclast maturation is driven by receptor activator of nuclear factor kappa-Β ligand (RANKL) and macrophage colony stimulating factor (M-CSF). Excessive osteoclast activation is linked to pathologic conditions, such as arthritis and osteoporosis [140]. Rheumatoid arthritis patients have increased arginine levels in the serum suggesting alterations in arginine metabolism [141]. L-arginine supplementation restrains inflammatory bone loss in experimental mouse arthritis and inhibits osteoclastogenesis in an ARG1-dependent manner tilting energy metabolism of inflammatory osteoclasts from glycolysis to oxidative phosphorylation [60, 141]. Conversely, arginine depletion by application of recombinant ARG1 also inhibits RANKL-driven osteoclastogenesis and ameliorates arthritis in mice [142]. These data suggest that both, supra- and hypophysiological amounts of arginine impair osteoclastogenesis. Hypoxia may induce iNOS expression in osteoclasts and promote osteoclastogenesis through NO-mediated effects [143, 144]. In accordance, iNOS deficient mice were reported to have bone abnormalities associated with reduced osteoclast-mediated bone resorption [145, 146]. NO also promotes osteoblast differentiation, as shown by ASL deficiency-mediated NO depletion [147]. Finally, protein citrullination by peptidylarginine deiminases (PAD) is essential for osteoclast differentiation and anti-citrullinated protein/peptide antibodies (ACPAs) are associated with bone loss and are therefore important diagnostic biomarkers in rheumatoid arthritis [148].

## The role of myeloid arginine metabolism in cancer

### Arginine metabolism in tumor-associated macrophages and tumor-associated neutrophils

Tumor-associated macrophages (TAMs) and tumor-associated neutrophils (TANs) infiltrate solid tumors, and control tumor growth and metastasis through various mechanisms, including T cell suppression, secretion of growth factors such as TGF-β, promotion of angiogenesis and matrix remodeling [149, 150]. Collagen taken up by TAMs is a rich source of arginine, which is further metabolized by ARG1 and iNOS [151–153]. Granulocyte–macrophage colony-stimulating factor (GM-CSF) produced by tumor cells and lactic acid synergistically induce ARG1 expression in TAMs [154]. The effect of GM-CSF was abrogated by inhibition of STAT3 and p38 MAPK, despite the fact that GM-CSF mainly signals via STAT5 [154, 155]. Moreover, the induction of ARG1 expression by lactic acid in combination with GM-CSF was mediated through the cAMP-cAMP response element–binding protein (CREB) pathway [154]. ARG1 expression generally correlates with the immunosuppressive function of TAMs and is dependent on arginine availability [154, 156]. For instance in glioma tumors, arginine deprivation reduces ARG1 expression in tumor-associated macrophages/microglia and enhances their proinflammatory and phagocytic function [156].

Similar to M2-like macrophages, ARG1-expressing TAMs and TANs deprive T cells from arginine thereby suppressing their anti-tumor effects [157, 158]. Extracellular L-arginine is required for the expression of T cell antigen receptor ζ chain (CD3ζ) and T cell proliferation [159, 160]. Consequently, ARG1 inhibition enhances the effects of immunotherapy, increases tumor-infiltrating CD8^+^ T cell and natural killer (NK) cell populations and reduces tumor growth in mice [161–163]. TANs also express ARG1 but in contrast to TAMs, do not metabolize arginine; instead they exocytose ARG1 in granules, which consumes arginine and thereby suppresses T and NK cell function [164–166]. Not only ARG1 but also iNOS mediates TAM immunosuppressive effects through production of peroxynitrite, which causes nitration and nitrosylation of the TCR and its co-receptor CD8 at tyrosine residues, thereby disrupting TCR signaling [167].

Tumor cells are auxotrophic for arginine [168]. They use arginine for protein synthesis and iNOS-mediated catabolism; in fact, tumor iNOS expression correlates with increased tumor cell proliferation, migration and metastasis [169–171]. Hence, competition of tumor cells with TAMs for arginine may also limit tumor growth [157]. In fact, to overcome arginine shortage, tumor cells can acquire the ability to upregulate arginine biosynthesis through the argininosuccinate shunt [172].

Moreover, TAMs produce high amounts of polyamines, which are associated with both, malignancy and immunosuppression [173]. TAM-derived polyamines may be taken up by tumor cells and promote their proliferation [173]. On the other hand, polyamines buffer the low intracellular pH in TAMs to support their survival in the acidic environment of solid tumors [174]. Consequently, simultaneous blockage of synthesis and uptake of polyamines reduces the myeloid/T cell ratio, decreases tumor growth in a CD8^+^ T cell-dependent manner and increases survival of tumor-bearing mice [174–176]. Finally, TAMs can produce creatine, which is taken up by tumor cells through the SLC6A8 transporter to promote their survival, while inhibition of creatine transport hinders tumor growth [177].

## Arginine metabolism in myeloid-derived suppressor cells

Myeloid-derived suppressor cells (MDSCs) are a heterogeneous population of rather immature, immunosuppressive myeloid cells, which are particularly abundant in cancer and chronic inflammatory diseases [178, 179]. Increased amounts of MDSCs correlate with poor prognosis in cancer patients [180]. They are classified into polymorphonuclear-(PMN)-MDSCs and monocytic-(M-)MDSCs [149, 181]. However, it remains unclear whether MDSCs are indeed a distinct immature myeloid cell type, as initially described, or merely myeloid cells, which gained immunosuppressive function in response to local conditions [182]. Arginine metabolism plays a pivotal role in the immunosuppressive function of MDSCs. ARG1 is highly expressed in human PMN-MDSCs thereby depriving T cells from arginine [183–185]. This restrains T cell proliferation and impairs T cell receptor CD3ζ chain expression, thereby suppressing Τ cell function [184]. While mouse MDSCs deplete arginine via its uptake and intracellular metabolism, human MDSCs mainly release arginase [186]. MDSCs may also secrete ARG1 within extracellular vesicles [187]. Consequently, released ARG1 decreases arginine levels in the local microenvironment impairing T cell function [78]. Accordingly, in cancer patients, arginase activity in PBMCs is strongly upregulated, while arginine amounts in plasma and expression of the CD3ζ TCR chain are diminished [188]. T cell-deriving cytokines, such as IL-4 and IL-10, can induce ARG1 expression in MDSCs [189]. Mechanistically, cyclooxygenase 1 and 2 (COX-1 and −2)–dependent PGE_2_ production by tumor cells promotes ARG1 expression in MDSCs in a paracrine fashion [190]. Moreover, β2 adrenergic signaling via STAT3 drives *Arg1* gene expression in MDSCs [191, 192]. Interestingly, administration of the pegylated form of the catabolic enzyme arginase I (peg-Arg I) blocked T cell proliferation and function, and induced MDSC accumulation through an eIF2a-dependent mechanism leading to increased tumor growth [193]. ARG1^+^ MDSCs release polyamines, which promote an immunosuppressive phenotype of dendritic cells via indoleamine 2,3-dioxygenase 1 (IDO1) activation [67]. However, MDSCs also inhibit CD8^+^ T cell function through direct cell-to-cell contact and NO-mediated nitration of TCR proteins causing TCR conformational changes [194]. Concluding, arginine metabolism is key for the immunosuppressive function of MDSCs. Ongoing clinical studies hence test its therapeutic targeting in cancer [195].

## Myeloid arginine metabolism in surgery

Surgical stress causes postoperative immunological changes including MDSC expansion and suppression of NK cell function, which in case of cancer surgery increases the risk of postoperative metastatic disease [196–201]. ARG1-expressing MDSCs lead to post-operative systemic depletion of arginine [202]. Consequently, arginine-deprived conditions lead to impaired NK cell proliferation, cytotoxicity and IFNγ production [203]. Perioperative arginine supplementation was shown to improve recovery, mitigate postoperative infections, accelerate NK cell recovery and reduce post-operative metastases [200, 201, 204].

## Concluding remarks

NO production and the argininosuccinate shunt promote inflammation and anti-microbial responses. In contrast, polyamine and proline metabolism is associated with anti-inflammatory and tissue-restorative responses. At the junction of these branches of arginine metabolism lies ARG1, which regulates the balance between pro- and anti-inflammatory myeloid responses. Dysregulation of this balance leads to non-resolving inflammation and tissue destruction as is the case in chronic inflammatory diseases, or to immunosuppression in cancer. Since arginine metabolism plays a critical role in myeloid cell reprograming, the therapeutic potential of targeting certain immuno-regulatory nodes in arginine metabolism may be leveraged to treat diseases, which are driven by dysregulated immune function.

## Data Availability

No original data was generated for this review article.
